# Why do my patients still have early childhood caries? a critical appraisal

**DOI:** 10.1590/1807-3107bor-2025.vol39.065

**Published:** 2025-07-07

**Authors:** Luciana Butini OLIVEIRA, Flávio de Freitas MATTOS, Saul Martins PAIVA, Marcelo BÖNECKER

**Affiliations:** (a) Faculdade São Leopoldo Mandic, Division of Pediatric Dentistry, Campinas, SP, Brazil.; (b) Universidade Federal de Minas Gerais – UFMG, School of Dentistry, Department of Public Health, Belo Horizonte, MG, Brazil.; (c) Universidade Federal de Minas Gerais – UFMG, School of Dentistry, Department of Pediatric Dentistry and Orthodontics, Belo Horizonte, MG, Brazil.; (d) Universidade de São Paulo – USP, School of Dentistry, Department of Pediatric Dentistry and Orthodontics, São Paulo, SP, Brazil.

**Keywords:** Oral Health, Evidence-Based Practice, Dental Caries, Child, Pediatric Dentistry

## Abstract

The prevalence of early childhood caries (ECC) tends to increase in the first 5 years of life as children grow, and those affected by ECC are likely to experience caries throughout their lives. ECC negatively impacts the oral health-related quality of life of both children and their families. Although controlling sugar intake and the use of fluoride are well-known methods for managing dental caries, preventive and therapeutic interventions alone have not been sufficient to prevent the development of new caries lesions. This review aims to explore why ECC continues to occur despite the oral health team having the necessary knowledge to prevent it. Based on current scientific evidence, this article highlights the need for the oral health team to consider additional factors, such as implementing oral health prevention programs in the first 450 days of life, enhancing caregivers’ oral health literacy, creating a supportive environment, and engaging in upstream actions (teledentistry, implementation science, health policies development etc.) to effectively manage and prevent dental caries. Otherwise we would be educating and treating children and sending them back to the conditions that made them sick. By addressing these factors, it might be possible to improve long-term oral health outcomes for children and reduce the burden of ECC on children and their families.

## Introduction

A systematic review using the World Health Organization (WHO) diagnostic criteria revealed significant variability in the prevalence of early childhood caries (ECC) across different regions of the world. The prevalence rates by continent were as follows: Africa at 30%, the Americas at 48%, Asia at 52%, Europe at 43%, and Oceania at 82%.^1^ The observed variance was primarily attributed to differences between countries rather than variations between continents or changes over time.

Globally, there are an estimated 532 million cases of untreated caries in deciduous teeth.^2^ Evidence suggests that one of the prevalence peaks of untreated caries occurs at the age of 6 years, primarily associated with ECC, while other peaks occur at 25 and 70 years of age.^3^ Regrettably, children affected by ECC tend to experience caries throughout their lives^4,5^ and are also more likely to report poorer self-rated general health in midlife.^6^

ECC prevalence tends to rise as children age during their first 5 years of life. According to the ECC Bangkok Declaration, the global prevalence of ECC in 1 year-old children has been is 17% while it increases to 63% at 5 years of age.^7^This pattern has also been observed in epidemiological studies in Brazil.^8-10^ However, there is a lack of updated or available estimates for the burden of ECC in certain Latin American and Caribbean countries.^11^

Oral diseases represent a substantial financial burden and are ranked as the fourth most expensive condition to treat.^12^ In the United States, surveys on medical expenditures revealed that dental costs for children under 5 years of age exceeded $1.55 billion dollars in 2010.^13^ Untreated ECC has already been associated with various negative outcomes, including impaired growth,^14^ nutritional deficiencies,^15,16^ behavioral and sleep issues,^17^ poor quality of life,^18^school absenteeism, and low educational performance.^19^ Furthermore, children with untreated ECC had significantly poorer oral health-related quality of life (OHRQoL) than children without ECC.^20,21^ Severe ECC with pulp involvement has a negative impact on the OHRQoL of both preschoolers and their families.^22-24^

The main risk factors for dental caries have been extensively studied in the dental literature, with past caries experience^25,26^ and social economic status among the most prominent ones. A recent prospective longitudinal study identified that higher severity of untreated dental caries, lower monthly income and higher B-ECOHIS scores were risk factors for the development of new untreated dental caries lesions.^27^ Systematic reviews have indicated that preschool children whose parents have a low education level were more likely to experience a greater increment in ECC over a 2-year period.^28^

Factors such as low maternal education, maternal age, low family income, parental education level, and low social class, have been identified as risk factors for ECC.^29^ It is important to consider the analysis of behavioral characteristics of the parents, such as resilience, a psychosocial factor that can affect health. Parents are the decision makers in their children’s lives, including the decision to seek dental treatment.^23^ A structural modeling unveiled that, in addition to lower socioeconomic status and lower parental resilience, higher consumption of free sugars was associated with greater ECC severity.^24^ This emphasis on the impact of frequent sugar consumption was corroborated by findings of a systematic review of case-control and cohort studies.^30^

Research shows a dose-dependent relationship between the consumption of free sugars and the onset of non-communicable diseases (NCDs), including dental caries, overweight, obesity, type 2 diabetes, and cardiovascular diseases.^31,32^ According to the WHO, free sugar is the fundamental dietary element contributing to the development of dental caries.^33^ Sugar triggers the dental caries process and the casual sequence of disease; in the absence of sugar, the causal sequence is interrupted and the onset of disease is prevented. Nonetheless, dental caries is a multifactorial condition, as the process also involves other biological, behavioral, and socioeconomic factors, but these factors mainly impact the speed of the cariogenic effects of sucrose or its consumption frequency.^34^

Fluoride toothpastes are particularly important in preventing and managing dental caries, and parents should use them daily to maintain their child’s oral health. The effectiveness of fluoride toothpastes in reducing the incidence of caries among preschool children is well established.^30,35^ Fluoride exposure delays the onset of dental caries lesions and the initiation of the cavitation, but its use as a standalone measure does not eliminate the risk of dental caries.

Enamel defects, especially hypomineralization, are also risk factors for dental caries, as reported in the systematic review by Kirthiga et al.^30^ in 2019. Epidemiological studies have shown that the prevalence of enamel defects in primary teeth ranges from 5.3%^36^ to 63.1%.^37^ Such defects facilitate the development of caries lesions due to the high enamel porosity, which favors dental plaque accumulation.^38-43^ Moreover, enamel porosity can lead to fractures or even structural breakdown, facilitating the accumulation of dental plaque and the development of caries lesions when sucrose is present.

In summary, the dental literature clearly shows that ECC can occur within the first year of life. It represents a significant financial burden for society and has a high impact on the quality of life of both children and their families. There is no doubt that dental caries is the most prevalent preventable chronic childhood disease, and studies have identified the main risk factors for ECC and the means to prevent it.

Despite the clinical evidence supporting the various preventive and therapeutic interventions against ECC, these interventions are not frequently applied in clinical settings. Moreover, oral health habits and access to care are influenced by the social determinants of health.^44^ The development of public policies for ECC prevention that address all the important factors involved in caries initiation and development are urgently needed. The aim of this review is therefore to thoroughly examine dental caries control from a standpoint firmly rooted in the latest scientific evidence and in the so-called ‘Dahlgren and Whitehead model’ of the main determinants of population health. In addition, we explore how primary teams, considering the various professional groups, can contribute to promoting oral health in children.

### How to tackle ECC from the perspective of the determinants of health?

Most oral conditions have a multifactorial etiology and are modulated by biological, social, economic, cultural, and environmental factors.^44^ The year 2021 marked the 30th anniversary of the Dahlgren and Whitehead’s model of the main determinants of health, sometimes referred to as the ‘rainbow model’. The model broadens horizons and encourages individuals to consider beyond health services and the health sector to address the broader social determinants of health within local environments and society. Based on this model, we need to find ways to better explain the vertical links between the social, economic, and cultural determinants of health and those of lifestyle. There is a need to advocate for a concerted action on the social determinants of health and the drivers of these determinants that lead to growing inequalities.^45^

### Strategies from a short-term perspective

Children who are told to brush their teeth with fluoride toothpaste and avoid sugary snacks may have economic constraints and be in school environments that do not support these health behaviors. For instance, toothbrushes and toothpastes might be available but expensive. Schools might not offer organized meals might not be available and sugary snacks are often sold by vendors and stores.^46^ In supermarkets, it is very common for unhealthy foods to be displayed at checkout counters.

Since our choices are largely shaped by the environment in which we live, a supportive environment makes it easier for individuals to choose healthier options. Reducing oral health disparities requires the implementation of effective and suitable policies to promote oral health, such as taxing sugary foods, exempting fluoride toothpastes, as helping people acquire the skills needed to make healthy choices.

A recent study carried out in Australia reported that many countries tax sugar-sweetened beverages (SSB), and this has shown to be effective in decreasing purchases of these beverages.^47^ Commercial lobbying is frequently a barrier to the development and implementation of public health policies.^48^ “Combating the commercial determinants of oral diseases and other non-communicable diseases should be a major policy priority”.^49^

Recently, the taxation of sugar-sweetened beverages^50^and its benefits in public health has been explored, and the potential synergies with other interventions make it an attractive option for policymakers.^51^ On the other hand, these data should be analyzed with caution. An umbrella review found that the effect of sugar-sweetened beverage taxation on sugar intake and dental caries is limited. A 20% volumetric tax on sugar-sweetened beverages would have a modest impact on the prevalence and severity of dental caries in high-, low-, and middle-income countries.^52^

Treatment alone will never effectively address the underlying cause of ECC. Disparities in preventive dental services for children still persist.^53-55^Caries risk assessment is key to establishing the probability of individual patients or groups of children developing carious lesions.^7^ Therefore, a more progressive approach to health promotion is required that acknowledges the significance of addressing the underlying social, political and environmental factors contributing to ECC.

In brief, in order to succeed in dental caries prevention, it is crucial to also consider oral health education, oral health literacy and a supportive environment. Otherwise we would be educating and treating the children, but sending them back to the conditions that made them sick in the first place.

### Strategies from a mid-term perspective

How can primary healthcare teams, considering the various professional groups, contribute to oral health promotion? Is the approach of oral health teams when working with children and their families based on health promotion or on the restorative-surgical model? It is important to mention that caries management by risk assessment is best practice as it is an evidence-based model that focuses on prevention and treatment of disease at the patient level rather than a restorative-surgical approach at the dental level.^56^

In order to reduce the prevalence and burden of ECC worldwide, the IAPD Bangkok Declaration^7^recommends that dentists, dental hygienists, physicians, nurses, health professionals, and other stakeholders be made aware of ECC and that preventive guidance within the first year of life be provided by a health professional or community health worker (building on existing programs - e.g. vaccination wherever possible), which should provide referral to a dentist for comprehensive continuing care. Professional organizations and interprofessional prenatal oral health guidelines (i.e., assess, advise, refer, share/coordinate) can prove beneficial to the community when it comes to practice behaviors.^57^

Although there is substantial and robust scientific evidence on effective preventive methods, regrettably, community-based initiatives in oral health promotion is either limited or underutilized in numerous countries.^12^

A transdisciplinary approach is essential for enhancing ECC prevention and to raise awareness of this condition among all health professionals.^5^Implementing a transdisciplinary approach to fight sugar consumption, a common risk factor for systemic and NCDs in children and adults, is currently considered the most effective strategy. Therefore, strategies on oral health promotion, prevention and treatment must be implemented into overall NCD policies.^58^Health professionals should enable people to make healthy choices.^5^Moreover, it is crucial to extend prevention and primary oral health care to schools and communities to effectively improve and promote oral health.^12^

Teledentistry is also an effective method for enhancing ECC prevention and improving population oral health.^59,60^ Asynchronous communication and the use of smartphones for image capturing can help in the implementation of teledentistry. Approaches based on new digital health technologies can contribute to better oral health for all. In the context of the Be He@lthy Be Mobile initiative, the World Health Organization and the International Telecommunication Union have developed “Mobile technologies for oral health: an implementation guide”.^61^ In addition, a guide was created for use by doctors, nurses, midwives, allied health professionals, and community health workers who have been formally trained and are registered with a relevant organization to provide health care to infants, children, and adolescents in various settings such as the community, primary care, and hospitals.^62^

Implementation science is essentially a set of scientific research methods designed to facilitate the integration of findings from scientific studies into the everyday practice of healthcare services. Its primary goal is to enhance community health by establishing clear objectives for the adoption of these research outcomes.^63^ However, there is a gap between evidence-based data and decision-making in clinical practice.^64-67^

A study regarding implementation science characteristics for a prenatal oral health e-health application provided vital information to facilitate the translation of the interprofessional prenatal oral health guidelines into clinical prenatal oral health practice.^68^

It is important to link public health policies to other policies. Recently, the WHO presented six guiding principles at the Global Action Plan for Oral Health 2023-2030. They are: a public health approach to oral health; integration of oral health into primary health care; innovative workforce models to respond to the oral health needs of the population; people-centered oral health care; personalized oral health interventions across the lifespan; and optimization of digital technologies for oral health. The plan contains the full set of policy documents that define the WHO’s global oral health agenda, which set out the path to address the challenges facing communities around the world, and advocate for the integration of oral health into NCDs and universal health coverage benefit packages.^69^

### Strategies from a long-term perspective

Due to the epidemiological of ECC, dental caries prevention should start within the first 1000 days of life or even earlier. Early feeding practices and promoting breastfeeding while avoiding sugar consumption should be embattled in the first 2 years of life.^70^

Dental caries prevention should start within the initial 450 days of life, encompassing 9 months of pregnancy and 6 first months of the child’s life, when the first tooth generally erupts in the child’s mouth. This period should be considered the best time to educate families about adopting healthy habits that can have a positive impact throughout the child’s life course.^5^

Family characteristics during pregnancy and early life were associated with caries experience in 5-year-old children.^71^ The BRISA cohort showed that being overweight or obese, as well as thin/very thin was associated with ECC in children, independent of socioeconomic variables and a high frequency of sugar consumption.^72^ Moreover, the cohort study demonstrated a correlation between the consumption of sugary drinks in pregnancy and maternal pre-gestational BMI (body mass index) and early exposure to products with high sugar content and BMI z-score in the second year of life. Maternal obesity and sugary drinks consumption in pregnancy increased the risk of early exposure (before to 2 years) and high exposure to added sugar, showing the perpetuation of unhealthy dietary behaviors in the first 1000 days of life.^73^ Findings of a prospective study among Scottish young children provide evidence that the introduction of sugar-sweetened beverages during the first year of life can put children in a trajectory of high levels of dental caries.^74^

To prevent ECC, it is recommended that a child first’s visit to the dentist should occur within the first 450 days of life, rather than the first 1000 days. Pregnancy is an ideal time to promote primary prevention of oral diseases in children and convey the oral-systemic connection given the profound influence of maternal health and behaviors on children’s oral health outcomes.^75^Scientific evidence related to the association between prenatal oral health care, ECC incidence, and Streptococcus mutans bearing in children showed a reduced ECC incidence and S. mutans bearing in children whose mothers received prenatal oral health care.^76^

To prevent ECC, members of the oral health team should provide the following to children and their families during the initial 450 days of life: raise awareness of ECC with parents/caregivers; limit sugar intake in foods and drinks and avoid free sugars for children under 2 years of age; brushing twice daily with fluoridated toothpaste (at least 1000ppm) for all children, using an age-appropriate amount of paste; provide preventive guidance within the first year of life.^58^

Even in cases where children and family have the opportunity to receive oral health education, new caries lesions can still occur, as education and information do not always lead to a transformation of a person’s behavior. There is no long-term evidence in respect of the effectiveness of oral health education on oral hygiene and dental caries in schoolchildren.^77,78^

Moreover, many people might have access to information and education in oral health, but lack the oral health literacy to understand the recommendations. Oral health literacy is the degree to which individuals have the capacity to obtain, process, and understand basic health information and represents an aspect of growing interest in the literature.^79^

A previous study assessed the relationship of oral health literacy (OHL) of parents on the decayed, missing, and filled teeth (DMFT) index of themselves and their children. There was a correlation between the OHL of the parents and the number of filled teeth in children. Children whose parents had adequate OHL had a significantly higher number of fillings and fewer missing teeth. Only 48.5% of the parents had adequate OHL.^80^

Caregiver’s OHL was associated with prevalence of untreated dental caries in preschool children.^81^ Another study investigated parents’ behavior during their children’s meals and their level of OHL. Parents with lower OHL had greater odds of having children with at least one clinical consequence of untreated dental caries than parents with higher OHL.^82^ Indeed, systematic reviews have shown that low OHL of parents was associated with dental caries in their children.^55,83^

How can we improve the work of oral health teams in improving oral health literacy? Oral health teams must be engaged in addressing the multiple aspects of the cause of caries. Engaging health team, families, community leaders, educators, and policy makers will help in the joint creation of a framework to be applied in and with the community.^84^The perspective of oral health care should be centered on the child and his family, taking into account his or her life context. Oral health education actions need to consider culture, economy, habits, values, and customs based on the realities of children and their families.

Delegating prevention and primary oral care to oral health auxiliaries and community health workers can help control caries in dental facilities, schools, and communities by improving and promoting oral health effectively.^12^

It is important to highlight that there is no point in having OHL if children do not have access to educational/preventive measures and treatments. Oral health education does not always lead to behavior change and many parents and caregivers lack OHL, thus the environment must be naturally supportive.

A supportive environment is necessary to empower children and their families to maintain behaviors that promote oral health. The delivery of health promotion strategies at the population level has shown a great impact on reducing the prevalence of oral diseases.^85^

The universal social gradient in both general and oral health highlights the underlying influence of psychosocial, economic, environmental, and political determinants. A conceptual shift is needed away from the biomedical/behavioral “downstream” approach to one addressing the “upstream” underlying social determinants of population oral health. Failure to change our preventive approach is a neglect of ethical and scientific integrity. A range of complementary public health actions may be implemented at local, national and international levels to promote sustainable oral health improvements and reduce inequalities.^86^

### Recommendations

There is available evidence supporting the effectiveness of methods for prevention of ECC such as: preventive dental programs for pregnant women; advice on diet and feeding; prenatal oral health care; integration of maternal and children’s oral health promotion into nursing practice; maternal oral health programs undertaken by non-dental health professionals; dental health education in combination with early preventive dental visits and the use of fluoride in children, which can be used in the form of toothpastes with more than 1000 ppmF. Fluoride varnishes can also be recommended according to the child’s needs.^87^

While dentists continue to work independently in their dental offices, striving for the oral health of their individual patients, there will be a portion of their patients who will develop new caries lesions. This approach is centered on a clinical practice with an emphasis on interventionism and high technology. Individualized care has a limited positive impact and only for the patient who receives it. This is a downstream action. Dentists should look to take more active roles in the community and try to somehow contribute to a healthier society, work at an upstream level.

The presence of a social disparity in oral health, including dental caries, requires policies and interventions aimed at guaranteeing that every child has equitable access to quality healthcare, a safe and healthy environment, life prospects, and health resources (social determinants of health).

An integral component involves addressing various layers, including infrastructure and service accessibility, as well as effecting fundamental alterations to economic, cultural, and environmental circumstances.

A recent systematic review investigated factors perceived by health professionals to be barriers or facilitators of caries prevention in children. The results showed that parents are another obstacle to children’s oral prevention. They may not recognize their child’s oral health as a priority due to their lack of knowledge, parenting skills, and health literacy. Additionally, health professionals might lack dental knowledge, self-confidence, and have an unclear understanding of their role in promoting oral health. Initiatives focused on improving professional oral health education are indispensable to improve communication between the oral health team and the family.^88^ Moreover, oral health education actions should be contextualized from the realities of children and their families. Oral health teams can improve oral health literacy, and public health policies should be linked to other policies.

## Data Availability

The content will be available when the article is published..
